# Reductive stress in cancer immunology and targeted therapy

**DOI:** 10.3724/abbs.2025173

**Published:** 2025-09-19

**Authors:** Xiaotian Ji, Gang Xiao

**Affiliations:** 1 Bone Marrow Transplantation Center of the First Affiliated Hospital and Institute of Hematology Zhejiang University School of Medicine Hangzhou 310058 China; 2 Liangzhu Laboratory and Institute of Immunology Zhejiang University Hangzhou 311121 China

**Keywords:** cancer, immunity, reductive stress, oxidation-reduction, reducing agent

## Abstract

Reductive stress is characterized by the excessive accumulation of cellular reducing equivalents, leading to the disruption of cellular redox homeostasis and a shift toward a reductive intracellular environment. Immune cells exhibit particularly dynamic redox modulation to adapt to activation and differentiation processes during immune responses, such as tumor recognition and destruction. Unlike their immune counterparts, tumor cells employ a specific metabolic mode for uncontrolled proliferation and survival, which may also lead to a shift in the intracellular redox balance. While extensive research has focused on oxidative stress during the immune response and cancer treatment, studies on reductive stress are still in their infancy. This review summarizes the generation process of reductive stress and its impact on cellular function, detailing its mechanisms in immune cells and various cancers, as well as its relevance to cancer treatment. The aim of this study is to explore new avenues for cancer immunotherapy from the perspective of reductive stress.

## Introduction

Redox homeostasis is a balance between oxidative and reductive biochemical reactions; it is necessary for maintaining the fundamental functionality of a cell and has a central role in human health. Disruptions in cellular redox homeostasis can cause various diseases, including cardiovascular diseases, diabetes, Alzheimer’s disease (AD), and cancers [ 1– 3] . Reactive oxygen species (ROS), which are byproducts of cellular metabolic activities, include superoxide anion radicals, hydrogen peroxides, and other oxygen-containing radicals. Owing to their unpaired electrons, ROS exhibit high oxidative reactivity. At low concentrations, ROS act as signaling molecules to regulate various biological processes in cells, whereas excessive ROS accumulation leads to cytotoxicity, referred to as oxidative stress. Under these conditions, ROS within the cell uncontrollably over-oxidize lipids, proteins, and nucleic acids, resulting in the formation of lipid peroxides, aberrantly modified proteins with thiol groups (such as disulfide bonds), and 8-oxo-guanine complexes [4]. These products cause irreversible damage to biomacromolecules and subsequently trigger various cellular death mechanisms, including senescence, apoptosis, autophagy, and ferroptosis [5].

Immunotherapy represents a distinct approach to conventional cancer treatments by which the immune system is activated to eliminate tumor cells consistently and effectively. However, the dysregulated metabolic activity of tumor cells often creates a unique tumor microenvironment (TME) that suppresses immune cell activation and infiltration, rendering the immune system incapable of eliminating tumor cells. Imbalances in redox homeostasis can differentially affect malignant progression, immune surveillance and even the formation of an immunosuppressive TME. Therefore, targeting redox imbalances represents a promising avenue for enhancing the efficacy of immunotherapy.

**Figure 1:**
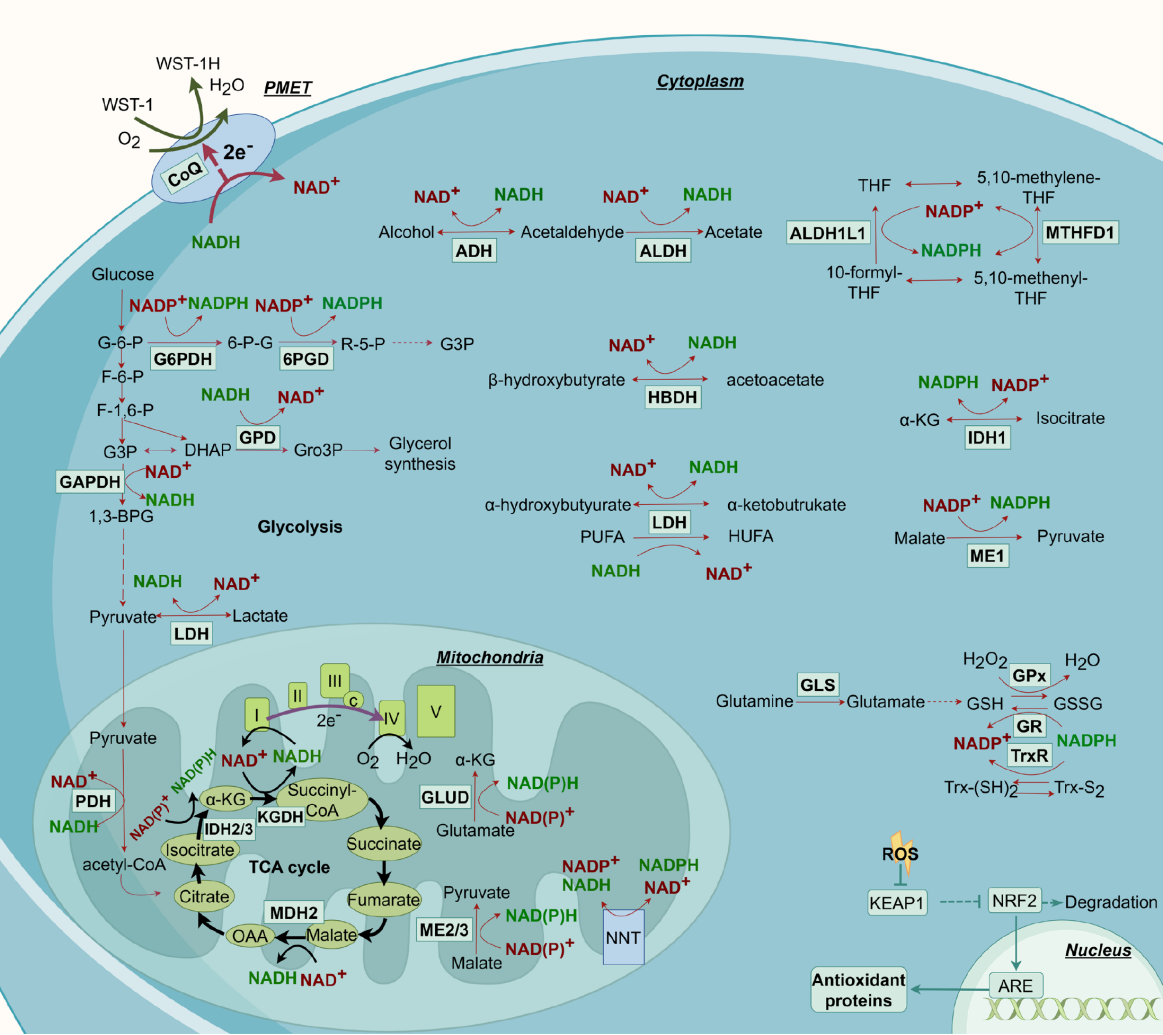
Figure 1 Reductants metabolism in redox homeostasis Both NAD(P)+ and NAD(P)H ensuring redox homeostasis within the intracellular environment and allowing cells to obtain sufficient energy and biosynthetic materials. GSH, Trx and NADPH serve as the reducing power within cells to power antioxidant enzymes and quench certain ROS. Gene expression systems like KEAP1/NRF2 system also play a vital role of antioxidant defense, regulating the expression of over 200 genes. Figure was created by Figdraw (https://www.figdraw.com).

Although oxidative stress in cancer has been extensively studied, the opposite of redox homeostasis—reductive stress—remains overlooked, despite its identification as a cause of cell death as early as 1989 [6]. Unlike oxidative stress, reductive stress is not solely defined by reduced ROS levels but rather by the excessive accumulation of reducing equivalents. The term “reducing equivalent” refers to the minimal molar amount of a substance that can react with an oxidizing agent in redox reactions. Its overaccumulation has been acknowledged as a hallmark of cellular reductive stress. This is exemplified by the increased ratios of nicotinamide adenine nucleotide hydrate (NADH)/nicotinamide adenine dinucleotide (NAD ^+^), nicotinamide adenine nucleotide phosphate (NADPH)/nicotinamide adenine nucleotide phosphate (NADP ^+^), and glutathione/glutathione disulfide (GSH/GSSG) ( Figure 1).

As previously discussed, the cellular reducing capacity comprises a diverse array of reductants integral to numerous metabolic pathways, each governed by distinct regulatory mechanisms. Consequently, reductive stress cannot be simplistically conceptualized as merely the inverse of oxidative stress, despite both disrupting redox homeostasis. Typically, reductive stress exerts detrimental cellular effects—manifesting as aberrant signaling, protein misfolding, mitochondrial dysfunction, and metabolic dysregulation—which culminate in suppressed proliferation or cell death [ 3, 7– 10] .

However, a paradoxical divergence emerges within cancer-immune dynamics. Both immune and tumor cells undergo metabolic reprogramming to fuel proliferation or differentiation within varying tissue microenvironments. Thus, reductive stress functions as a context-dependent modulator of cancer-immune interactions, and its net impact varies with tumor type, metabolic niche, and immune cell subset. It can simultaneously inhibit tumor cell proliferation, impair the immunosuppressive function of regulatory T cells (Tregs), and enhance infiltration by macrophages. Conversely, under specific conditions, reductive stress may confer chemoresistance to tumor cells and foster an immunosuppressive microenvironment.

This review summarizes recent advances in reductive stress research and analyzes its regulatory mechanisms, pathological relevance, effects on the immune system, and potential applications in cancer treatment. By elucidating the role of reductive stress in immune and tumor cells, we aim to broaden the scope of immuno-oncology and explore novel therapeutic strategies.

## Redox Homeostasis

Redox homeostasis refers to the state in which the levels of oxidants and reductants within cells are maintained to sustain various redox reactions. Chemically, it represents the balance between electron donors and acceptors. In redox reactions, reductants donate electrons, whereas oxidants accept them. Both reductants and oxidants serve as cofactors or substrates in enzymatic and non-enzymatic reactions in cells, coordinating electron transfer. To maintain continuous biological activities, cells need to maintain a certain proportion of oxidants and reductants in their internal environment, and the balance achieved between them is known as redox homeostasis. In mammalian cells, exogenous oxidants are often required for energy metabolism, and endogenous reductants in the cell perform antioxidant functions. Excessive oxidant accumulation shifts the intracellular environment toward oxidation, whereas exaggerated reductants tip the balance toward reduction, and fluctuations toward either direction disrupt redox equilibrium.

Over the course of evolution, mammalian ancestors developed aerobic respiration, efficiently producing energy-rich adenosine triphosphate (ATP) by consuming natural oxidant oxygen molecules. However, during energy metabolism and biosynthesis, some electrons leak onto oxygen molecules, generating a byproduct named reactive oxygen species [11]. These species are highly reactive and can oxidize proteins, lipids, and other biomolecules. Therefore, cells need an adequate supply of reductants to exert antioxidant effects and prevent the accumulation of excessive oxidants, including ROS, reactive nitrogen species (RNS) [12], and reactive sulfur species (RSS) [13]. ROS, RNS, and RSS are collectively referred to as highly oxidizing chemical substances derived from oxygen, nitrogen, and sulfur, respectively.

To counteract the excessive accumulation of oxidants, cells have evolved a complex redox regulatory network composed of antioxidant small molecules, vitamins, the thioredoxin (Trx) system, antioxidant enzymes, and gene expression systems that regulate redox homeostasis. GSH has a reducing thiol group and can be easily oxidized under the enzymatic activity of glutathione peroxidase (GPX) to glutathione disulfide (GSSG), a process that often reduces intracellular hydrogen peroxide (H _2_O _2_) and lipid peroxides. Conversely, GSSG can be reduced back to GSH by glutathione reductase (GR) at the expense of one molecule of reduced NADPH [14]. Similarly, vitamin C scavenges cellular ROS by being oxidized to ascorbate free radicals and dehydroascorbate [15]. Reduced Trx can oxidize itself to convert disulfides of target proteins to thiols. Trx reductase (TrxR) can consume NADPH to regenerate reduced Trx from oxidized thioredoxin disulfide, and reduced Trx can also generate reduced peroxiredoxin (Prdx) to release its reductase activity [ 16, 17] . Superoxide dismutase (SOD) converts superoxide anion radicals (O _2_^–^) to non-radical H _2_O _2_ [18], which is then reduced to water and oxygen by catalase (CAT) [19] or is reduced to water by Prdx. In addition to biochemical networks that counteract oxidative stress, transcription regulation is also involved in antioxidant defense. The transcription factor nuclear factor E2-related factor 2 (NRF2) plays a pivotal role in regulating the expression of more than 200 genes, which function mainly in neutralizing ROS, detoxifying, repairing DNA, and regulating inflammation [20]. Under redox homeostasis, NRF2 is bound by Kelch-like ECH-associated protein 1 (KEAP1) or β-transducin repeat-containing protein (βTrCP) and degraded by the proteasome. However, when excessive ROS accumulate in the cell, oxidized KEAP1 disassociates from NRF2, stabilizes NRF2 in the nucleus, and recognizes antioxidant response element (ARE) sequences to activate the transcription of protective genes, which could accelerate the production of reducing equivalents [21] ( Figure 1).

NAD ^+^ is an oxidizing cofactor that can be synthesized *de novo* in mammals and participates in a variety of enzymatic reactions [22]. NAD ^+^ can be phosphorylated by NAD ^+^ kinase (NADK) to form NADP ^+^, and nicotinamide nucleotide transhydrogenase (NNT) on the mitochondrial inner membrane can directly interconvert NADH and NADPH [23]. Both NAD ^+^ and NADP ^+^ can be converted to their reduced forms, NADH and NADPH, by corresponding dehydrogenases. NAD(P) ^+^ and NAD(P)H are involved in various metabolic pathways within the cell, such as glycolysis, the pentose phosphate pathway (PPP), and the tricarboxylic acid cycle (TCA cycle). During glycolysis, glyceraldehyde-3-phosphate dehydrogenase (GAPDH) converts glyceraldehyde-3-phosphate (G-3-P) to 1,3-bisphosphoglycerate, reducing NAD ^+^ to NADH. Ethanol dehydrogenase and aldehyde dehydrogenase in the cytoplasm also reduce NAD ^+^ to form NADH [24]. In the mitochondria, during the TCA cycle, pyruvate dehydrogenase (PDH) catalyzes the decarboxylation of pyruvate to acetyl-CoA, reducing NAD ^+^ to NADH. Other enzymes with similar functions include isocitrate dehydrogenase 3 (IDH3), α-ketoglutarate dehydrogenase (KGDH), and malate dehydrogenase 2 (MDH2). Additionally, malic enzyme (ME2) and glutamate dehydrogenase (GLUD1/2) in the mitochondria also generate NADH [25]. Conversely, lactate dehydrogenase (LDH) reduces pyruvate to lactate while oxidizing NADH to regenerate NAD ^+^ [26]. Mitochondrial NADH is oxidized back to NAD ^+^ by the electron transport chain (ETC) to synthesize ATP, which consumes oxygen and produces water. NADPH in the cytoplasm is derived mainly from the PPP pathway and is synthesized by glucose-6-phosphate dehydrogenase (G6PD) and 6-phosphogluconate dehydrogenase (6PGD), which reduce NADP ^+^ to NADPH. Moreover, cytosolic IDH1, ME1, methylenetetrahydrofolate dehydrogenase (MTHFD1), and aldehyde dehydrogenase 1 family member L1 (ALDH1L1), and mitochondrial IDH2, ME3, MTHFD2, and ALDH1L2 can also synthesize NADPH [27] ( Figure 1). The oxidized NAD(P) ^+^ and reduced NAD(P)H can be synthesized *de novo* and interconverted, ensuring redox homeostasis within the intracellular environment and allowing cells to obtain sufficient energy and biosynthetic materials.

Cellular exposure to hypoxia or pharmacological inhibition of reducing-energy-consuming processes precipitates the pathological accumulation of reductants that cannot be fully oxidized during OXPHOS or nucleotide and fatty acid synthesis. This electron burden disrupts intracellular redox homeostasis, thereby inducing reductive stress that manifests cytopathological consequences. However, research on reductive stress is still limited, and we need to understand the characteristics and effects of reductive stress from the perspective of metabolic fluctuations and their subsequent pathological relevance.

## Reductive Stress and Cellular Metabolism

The synthesis of various building blocks and bioenergetic processes within cells require the participation of oxidized NAD(P) ^+^ and reduced NAD(P)H. For example, NADP ^+^ acts as a cofactor for glucose-6-phosphate dehydrogenase (G6PD) to promote the pentose phosphate pathway (PPP), which produces ribose-5-phosphate as the precursor for nucleotide synthesis. NADPH is required for the synthesis of fatty acids (such as palmitic acid, cholesterol, sphingolipids, phospholipids, and triglycerides) that consume fatty acyl-CoA [22]. NADPH is also a necessary substrate for ribonucleotide reductase (RNR) during nucleic acid synthesis [28]. To supply the energy required for biosynthetic activities, NAD ^+^ acts as a cofactor for GAPDH to promote glycolysis, and NADH serves as an electron donor for the oxidative phosphorylation (OXPHOS) reaction. When certain metabolic pathways or regulatory networks are distorted to produce overwhelming reducing equivalents, the redox balance is disrupted, and both anabolic and catabolic processes can be impaired, resulting in significant cellular damage.

The two most common causes of reductive stress are hypoxia-induced cell respiratory blockage and overactivation of the KEAP1-NRF2 antioxidant system. For example, in lung fibroblasts under hypoxic conditions, the electron transport process of the respiratory chain on the mitochondrial inner membrane is blocked, leading to an increased NADH/NAD ^+^ ratio. Consequently, the TCA cycle is inhibited within the cell because of the overaccumulation of NADH, and the accumulated α-ketoglutarate in the cycle is reduced to 2-hydroxyglutarate (L2HG) by malate dehydrogenase [29]. Therefore, the accumulation of L2HG within the cell can reflect the reductive stress caused by hypoxia, mitochondrial defects, and TCA cycle blockage. Similarly, when cells are under reductive stress due to hypoxia or defects in the electron transport chain, the intermediate product of glycolysis, dihydroxyacetone phosphate (DHAP), is converted to glycerol-3-phosphate (Gro3P) in the cytoplasm by glycerol-3-phosphate dehydrogenase (GPD), which consumes NADH to generate NAD ^+^ and thereby alleviates reductive stress; this process is referred to as the glycerol-phosphate shuttle [30]. Thus, the increase in Gro3P content can also reflect the reductive stress status of the cell. The Gro3P synthesized in this process can be used for the synthesis of fatty acids and glycerol and can also be converted back to DHAP by Gro3P dehydrogenase within the mitochondria, providing electrons for the ETC to maintain ATP synthesis [30]. During oxidative stress, the KEAP1-NRF2 pathway functions to reduce ROS and counteract cellular toxicity. However, excessive activation of NRF2 can also lead to reductive stress in the cell [21]. For example, cells with defective autophagy accumulate p62 and further sequester KEAP1, consequently resulting in aberrant NRF2 translocation to the nucleus, causing reductive stress.

Considering the role of NADH/NAD ^+^ as cofactors for various metabolic reactions, it is not surprising to observe that the activities of certain metabolic steps are highly coordinated with redox status. In liver cells, the substrate-to-product concentration ratios of various dehydrogenases can reflect variations in the NADH/NAD ^+^ ratios across different intracellular compartments. For example, a higher ratio of lactate to pyruvate for lactate dehydrogenase indicates a higher ratio of NADH/NAD ^+^ in the cytoplasm. In addition to LDH, another metabolic pathway that promotes the recycling of NAD ^+^ and the synthesis of highly unsaturated fatty acids (HUFAs) from polyunsaturated fats (PUFAs) catalyzed by Δ-5 and Δ-6 desaturases (D5D/D6D) involves the use of NADH as an electron donor. This fatty acid synthesis pathway is highly inversely related to lactate fermentation, which could be a temporal adaptation when aerobic respiration is impaired with high NADH levels [31]. Similarly, the higher ratio of hydroxybutyrate to acetoacetate for β-hydroxybutyrate dehydrogenase suggests a higher ratio of NADH/NAD ^+^ in the mitochondria [32].

Owing to the detrimental impact of reductive stress, cells also possess anti-reductive regulatory mechanisms. When cells are under reductive stress due to continuous activation of the antioxidant regulatory system or hypoxia-induced blockage of respiration, the number of oxidized cysteine residues on estrogen receptor-interacting protein 1 (FNIP1) is reduced. This allows the E3 ubiquitin ligase CUL2 to recognize FNIP1 and ubiquitinate it for degradation. With the degradation of FNIP1, its inhibitory effect on mitochondrial activity is lifted, and the mitochondria are reactivated to produce ROS, thereby balancing the excessive electron donor within the cell. Knockout of *FNIP1* can rescue myogenic differentiation inhibited by reductive stress. Knockout of the CUL2 adaptor fem-1 homologue B (FEM1B) can also mitigate excessive reducing equivalents [ 33, 34] .

In summary, the reductive intracellular environment can be sensed and antagonized by cells through metabolic pathways that are coupled with the conversion of reductive and oxidative cofactors such as NAD(H). If excessively accumulated reducing equivalents cannot be oxidized, multiple cellular processes, such as energy production, signal transduction and proteostasis, can be impaired. Therefore, when normal cells are under reductive stress, various diseases develop.

## The Association of Reductive Stress with Various Diseases

Numerous studies have shown that reductive stress is associated with a variety of human diseases. The excessive accumulation of reducing equivalents in cells impedes signal transduction, interferes with the formation of disulfide bonds in proteins, and further induces protein misfolding, transport disorders, and aberrant aggregation, all of which can lead to disease [7].

Oxidative stress is commonly believed to be closely related to cardiovascular diseases. However, when heat shock protein 27 (HSP27), which has antioxidant properties, is highly expressed in mouse cardiomyocytes, the level of GPX1 is increased, leading to an increase in the GSH/GSSG ratio and reductive stress, which can also induce cardiomyopathy [1]. Shanmugam *et al*. [35] reported that when Nrf2 was highly expressed in mouse cardiomyocytes, the cells were under reductive stress, which induced heart failure. Zhang *et al*. [36] induced the synthesis of large amounts of GSH in embryonic rat cardiomyocytes via treatment with the antioxidant N-acetylcysteine, leading to reductive stress. This ultimately results in cardiomyocyte mitochondrial dysfunction and heart disease. An increased ratio of NADH/NAD ^+^ is closely related to myocardial ischaemia-reperfusion injury, and reductive stress is also an important pathological factor in coronary artery disease and myocardial infarction [37]. The overexpression of antioxidant enzymes (such as Prdx and GPX) can increase the intracellular content of GSH, resulting in ROS deficiency and reductive stress. Consequently, cardiovascular cells block growth factor-mediated signaling, leading to mitochondrial dysfunction and apoptosis, compromising angiogenesis and accounting for the pathogenesis of cardiovascular diseases such as atherosclerosis [8].

In addition to cardiovascular diseases, reductive stress has also been found to be involved in the occurrence and development of AD, mitochondrial diseases, steatotic liver diseases, chronic hyperglycemia, and male infertility. Badía *et al*. [2] examined young susceptible individuals carrying the *APOE4* allele, which is the main genetic risk factor for AD, and reported that their lymphocytes presented an elevated GSH/GSSG ratio, indicating reductive stress. In contrast, immune cells from patients with established AD exhibit characteristics of oxidative stress. These findings suggest that immune cells under reductive stress may be involved in the early stages of AD development. Moreover, a multi-omics analysis of patients with mitochondrial encephalomyopathy, lactic acidosis, and stroke-like episodes (MELAS) revealed that the occurrence and development of various mitochondrial diseases are directly related to an elevated NADH/NAD ^+^ ratio [10]. Mitochondrial reductive stress in hepatocytes is also a common marker of steatotic liver disease [38]. The activation of NRF2, which leads to excessive accumulation of GSH, can induce reductive stress and affect the proliferation and differentiation of skeletal muscle cells, thus inhibiting myofiber formation [39]. In hepatocytes and pancreatic β-cells of patients with chronic hyperglycemia, excessive accumulation of NADH during the TCA cycle can induce reductive stress. Consequently, the electron transport chain cannot effectively process excessive NADH, leading to electron leakage and the generation of ROS, which paradoxically induces oxidative stress. Under the combined effects of continuous redox imbalance, insulin resistance, and impaired insulin secretion, patients gradually develop chronic hyperglycemia [3]. During the antioxidant treatment of male infertility, a specific dietary structure can lead to the excessive accumulation of reducing equivalents, inducing reductive stress. The excess NADH then leads to leakage of electrons to generate ROS, which can damage DNA and adversely affect treatment outcomes [40].

In brief, reductive stress can cause dysfunctions in multiple tissues and organs of the human body and lead to the occurrence of various diseases. Reductive stress can paradoxically cooperate with oxidative stress in different stages of the same disease. Considering the involvement of the immune system in the development of various types of health conditions, it is not surprising that reductive stress exerts a substantial influence on the functionality of the immune system.

## The Impact of Reductive Stress on Immune Cells

Immune cells undergo profound metabolic reprogramming in response to dynamic redox fluctuations—a necessity driven by their functional demands for proliferation, differentiation, and synthesis of effector molecules (including complement proteins, cytokines, and antibodies). This metabolic plasticity is exemplified by the oxidative burst in neutrophils and macrophages, where rapid redox shifts enable pathogen clearance. When immune cells experience reductive stress due to genetic mutations or external conditions such as hypoxia, various immune functions can be impaired. In severe cases, reductive stress can lead to tumor immune evasion or prolonged inflammatory responses.

**Figure 2:**
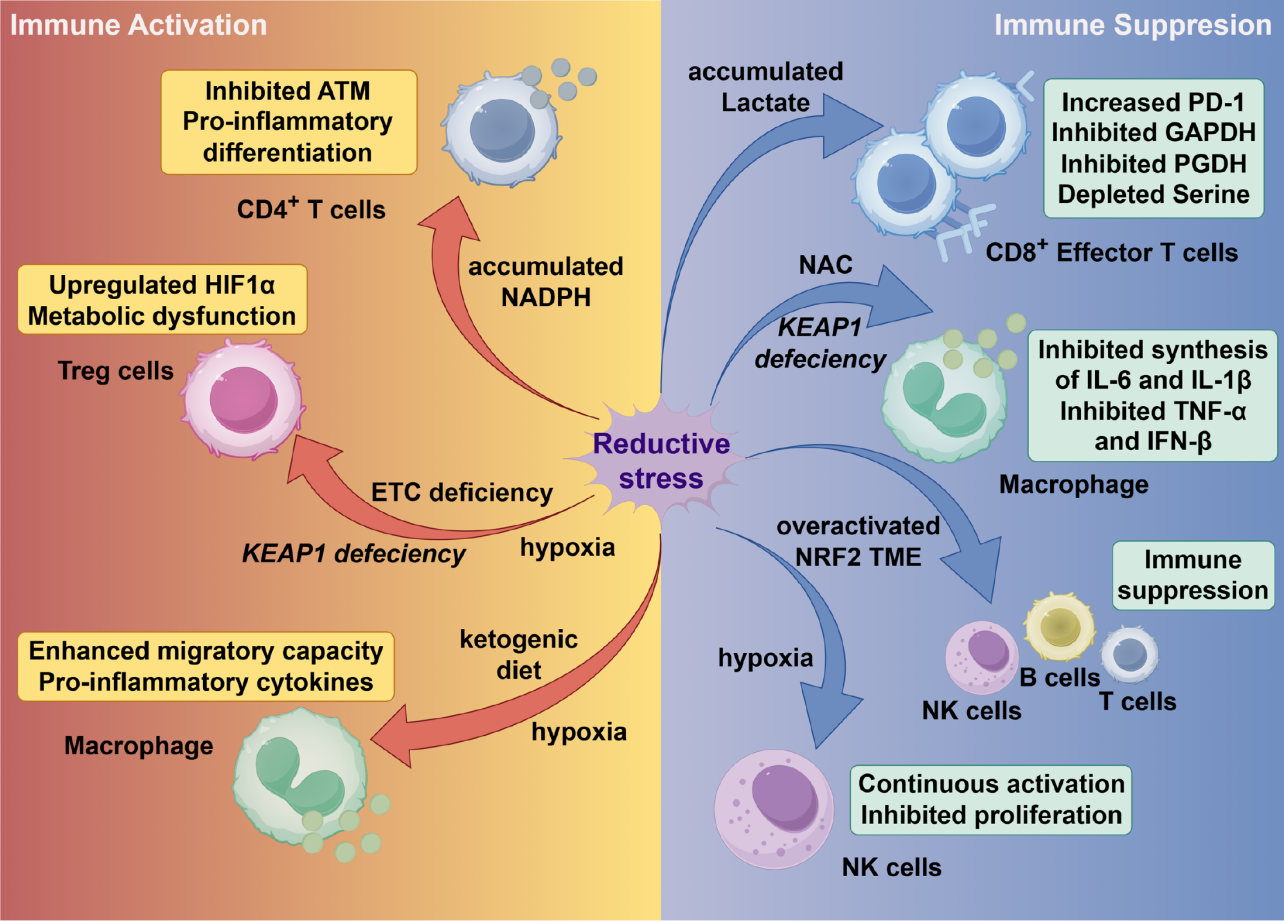
Figure 2 Reductive stress in immune cells The migratory capacity of macrophage would be enhanced due to reductive stress. The immune function varies under different reductive stress conditions. Reductive stress can inhibit immunosuppression function of Tregs, enhance migratory capacity of macrophages and induce pro-inflammatory characteristics. It will inhibit the proliferation of CD8+ effector T cells and contribute to formation of immunosuppressive TME. Figure was created by Figdraw (https://www.figdraw.com).

T cells reside in or function in various tissues and organs of the human body and adapt to diverse metabolic environments. The oxygen level varies in different tissues and organs, and T cells may encounter a hypoxic microenvironment. Metallo *et al*. [41] reported that hypoxic CD8 ^+^ T cells experience blocked mitochondrial respiration and reductive stress but promote proliferation by enhancing the reductive carboxylation of glutamine. Effector T cells in the placenta, gastrointestinal tract, and tumor microenvironment often exist under conditions of low glucose and high lactate levels. The large amounts of acquired lactate are reduced to pyruvate at the expense of the accumulation of NADH by lactate dehydrogenase in T cells, leading to reductive stress. The activities of GAPDH and phosphoglycerate dehydrogenase (PGDH), which depend on NAD ^+^ in the glycolytic pathway, are subsequently inhibited. Moreover, serine, a key metabolite for T-cell proliferation, is depleted because of impaired *de novo* synthesis, and effector T-cell functions are subsequently diminished ( Figure 2). Exogenous supplementation with serine can reverse the reductive stress induced by excessive lactate and restore the proliferative capacity of T cells [42]. Weyand *et al*.[ 43, 44] reported that the CD4 ^+^ T cells of rheumatoid arthritis patients exhibit metabolic reprogramming with inhibited glycolysis and an enhanced pentose phosphate pathway. Consequently, the large amounts of NADPH accumulated in the cells lead to reductive stress, and the reduced levels of ROS prevent the activation of ataxia-telangiectasia mutated proteins (ATMs), which sense that the ROS level changes. As a result, T cells differentiate into pro-inflammatory subsets that secrete interferon-γ (IFN-γ) and IL-17, causing severe inflammation. In hypoxic environments, Tregs upregulate the level of hypoxia-inducible factor 1α (HIF-1α) and experience reductive stress. Moreover, oxidative phosphorylation activity in Tregs is blocked, glycolysis is promoted, and their immunosuppressive function is inhibited. Genetic depletion of HIF-1α significantly promotes the ability of Tregs to inhibit CD8 ^+^ T cells [45]. Weinberg *et al*. [46] reported that when Tregs have a defect in mitochondrial electron transport chain complex III, blocked oxidative respiration leads to excessive accumulation of NADH and reductive stress. Under these conditions, Tregs lose their immunosuppressive capacity, ultimately leading to fatal inflammatory diseases and death in mice.

Changes in metabolic status can also induce reductive stress in macrophages. Hypoxic environments also induce macrophages to secrete large amounts of pro-inflammatory cytokines, such as interleukin 1β (IL-1β) and tumor necrosis factor α (TNF-α), promoting inflammation and enhancing the migratory capacity of macrophages [47]. A ketogenic diet can induce a shift towards reductive stress in the intracellular environment. Panico *et al*. [48] rapidly increased serum β-hydroxybutyrate levels in rats by administering 1,3-butanediol, which led to an elevated NADH/NAD ^+^ ratio and reductive stress, enhancing the ability of macrophages to infiltrate white adipose tissue ( Figure 2).

In addition to metabolic regulation, genetic alterations may also cause excessive accumulation of reducing equivalents. Klemm *et al*. [49] reported that genetic disruption of *KEAP1* in Tregs activated NRF2 and induced reductive stress, which inhibited the function of Tregs, increased the activity of effector T cells, and led to pulmonary immune cell infiltration, causing autoimmune inflammatory diseases. Kobayash *et al*. [50] reported that when the antioxidant system transcription factor NRF2 is highly activated when *KEAP1* is knocked out in macrophages, it induces reductive stress and immune response deficiency. Under these conditions, NRF2 in the cell nucleus inhibits the recruitment of RNA polymerase II, preventing macrophages from synthesizing the pro-inflammatory cytokines IL-6 and IL-1β after stimulation with lipopolysaccharide (LPS). Similarly, treatment with the antioxidant NAC also inhibits the secretion of TNF-α and IFN-β by macrophages after LPS stimulation [47]. Cunha *et al*. [51] reported that when NADPH oxidase 2 (NOX2) in macrophages is deficient or pharmacologically inhibited with apocynin (AP-O), the reductant Trx accumulates excessively in the cell nucleus after LPS stimulation, causing reductive stress. In addition, reductive stress increases the transcription of inflammatory factors through nuclear factor-κB (NF-κB), leading to chronic granulomatous disease and sepsis. Inhibiting TrxR can prevent the over-activation of NF-κB and prevent the excessive synthesis of TNF-α. Moreover, the reductive stress caused by the excessive accumulation of Trx also leads to increased activity of the nucleotide-binding domain and leucine-rich repeat pyrin-domain containing protein 1 (NLRP1) in macrophages, making the inflammasome more easily activated and triggering pyroptosis [ 52, 53] .

In summary, reductive stress has a significant effect on immune cells. It can inhibit the immunosuppressive activity of Tregs and increase the infiltration capacity of macrophages, although their ability to synthesize pro-inflammatory cytokines may vary under different conditions. For effector T cells, the impact of reductive stress depends on the specific context of pathogenesis. The most successful application of immunotherapy in recent decades has been accomplished in treating cancer patients. Many studies on the unique metabolic behavior and specific interactions of infiltrating immune components with tumor cells have been published. Characterizing and leveraging reductive stress in cancer immunotherapy is of particular interest.

## The Role of Reductive Stress in the Antitumor Response of Immune Cells

Tumor cells often interact with surrounding stromal cells, immune cells, blood vessels, and the extracellular matrix to form a unique tissue structure, namely, the tumor microenvironment (TME), which is often accompanied by aberrant environmental cues that induce reductive stress, such as hypoxia. Thus, immune cells are also affected by reductive stress during antitumor immune responses.

The distorted metabolic activities of tumors can also alter the composition of environmental metabolites, thereby altering the redox homeostasis of immune cells in the TME. Many malignant tumors, such as gliomas [54] and breast carcinomas [55], rely on glycolysis to synthesize energy substances and cellular components, resulting in the secretion of large amounts of lactate, which contributes to reductive stress in T cells. Mishra *et al*. [56] also reported that, in patients with squamous cell carcinoma, high expression levels of LDHA in tumor cells and LDHB in immune cells predict poor clinical outcomes.

In lung adenocarcinoma cells, overactivation of NRF2 promotes the formation of an immunosuppressive TME. In mice with *Keap1*-mutant lung adenocarcinoma, the numbers of NK cells, B cells, and T cells in the pulmonary tumor microenvironment are sharply reduced. CD8 ^+^ T cells express high levels of programmed death receptor 1 (PD-1), and the expression of programmed death receptor ligand 1 (PD-L1) on tumor cells also increases [57] ( Figure 2). The hypoxic environment of tumors also induces the continuous activation of dynamin-related protein 1 [58], decreasing the long-term survival and cytotoxicity of NK cells. Tumor cells, such as neuroblastoma [59] and glioblastoma [60] cells, often undergo metabolic reprogramming to deplete glutamine from the TME into tumor cells. Low levels of GSH synthesis due to glutamine deficiency further impair the cytotoxic function of NK cells [61].

As the key amino acid involved in redox balance, glutamine deficiency has also been reported to impede the activation of γδT cells, preventing them from exerting immune functions [62]. The reduced concentration of glutamine in the tumor microenvironment also inhibits the activation of B cells and leads to tumor immune evasion [63]. In an experimental model with a restricted glutamine supply, Martíne *et al*. [64] reported that TME CD4 ^+^ T helper 1 (Th1) cells, which activate CD8 ^+^ cytotoxic T cells to eliminate tumor cells, are displaced by Th2 cells that interfere with or even repress antitumor responses, which also indicates that glutamine deficiency caused by reduced tumor metabolism contributes to the formation of an immunosuppressive TME.

Jiang *et al*. [65] reported that transition metal borides, which have dehydrogenase-like activity, can cause excessive accumulation of cellular NAD(P)H and GSH, leading to reductive stress in mammalian cells. By using these transition metal borides in a mouse model of breast cancer, induced reductive stress creates an immunosuppressive microenvironment, increasing Treg infiltration and M2 macrophage differentiation, promoting the synthesis of immunosuppressive cytokines such as IL-4, IL-10, and transforming growth factor-β (TGF-β), and high expression of T-cell immunoglobulin mucin-3 (TIM-3) in T cells, which promotes tumor cell immune evasion and ultimately increases the ability of breast cancer cells to metastasize to lung tissue. In addition, the hypoxic environment also promotes the tumor-infiltrating capacity of Tregs [66], despite reports of the Treg-inhibiting role of HIF-1α [67].

These results indicate that reductive stress may promote the formation of an immunosuppressive TME, leading to cancer immune evasion. However, Renken *et al*. [68] reported the opposite phenomenon in melanoma patients. Treatment with auranofin, a potent activator of the transcription factor NRF2, enhances the cytotoxic capacity of tumor-infiltrating lymphocytes (TILs), NK cells from healthy donors, and chimeric antigen receptor (CAR) T cells targeting CD19 ^+^ tumor cells or autologous tumor spheroids ( Figure 2). Li *et al*. [69] also reported that when gastric cancer model mice were orally administered exogenous glutamine, elevated GSH levels in the plasma led to a more reducing tissue microenvironment, which improved the cytotoxicity of NK cells and significantly promoted the secretion of IL-2, thereby suppressing tumor growth *in vivo*.

In summary, the complexity of the tumor microenvironment generates a unique redox milieu for immune cells in a context-specific manner to affect the interaction between and infiltrating immune cells. In most cases, tumor cells under reductive stress appear to further promote the formation of an immunosuppressive microenvironment and facilitate immune evasion. However, in melanoma patients, a reduced TME promotes an antitumor immune response. Therefore, exploring the role and regulatory mechanisms of reductive stress in different types of cancer may provide new avenues for cancer immunotherapy.

## Reductive Stress in Cancers

The reprogrammed metabolic activities of tumors also lead to an imbalance in intracellular redox homeostasis, providing a new therapeutic strategy by exacerbating this imbalance. While there has been extensive research on oxidative stress in cancer, the effect of reductive stress remains vague.

**Figure 3:**
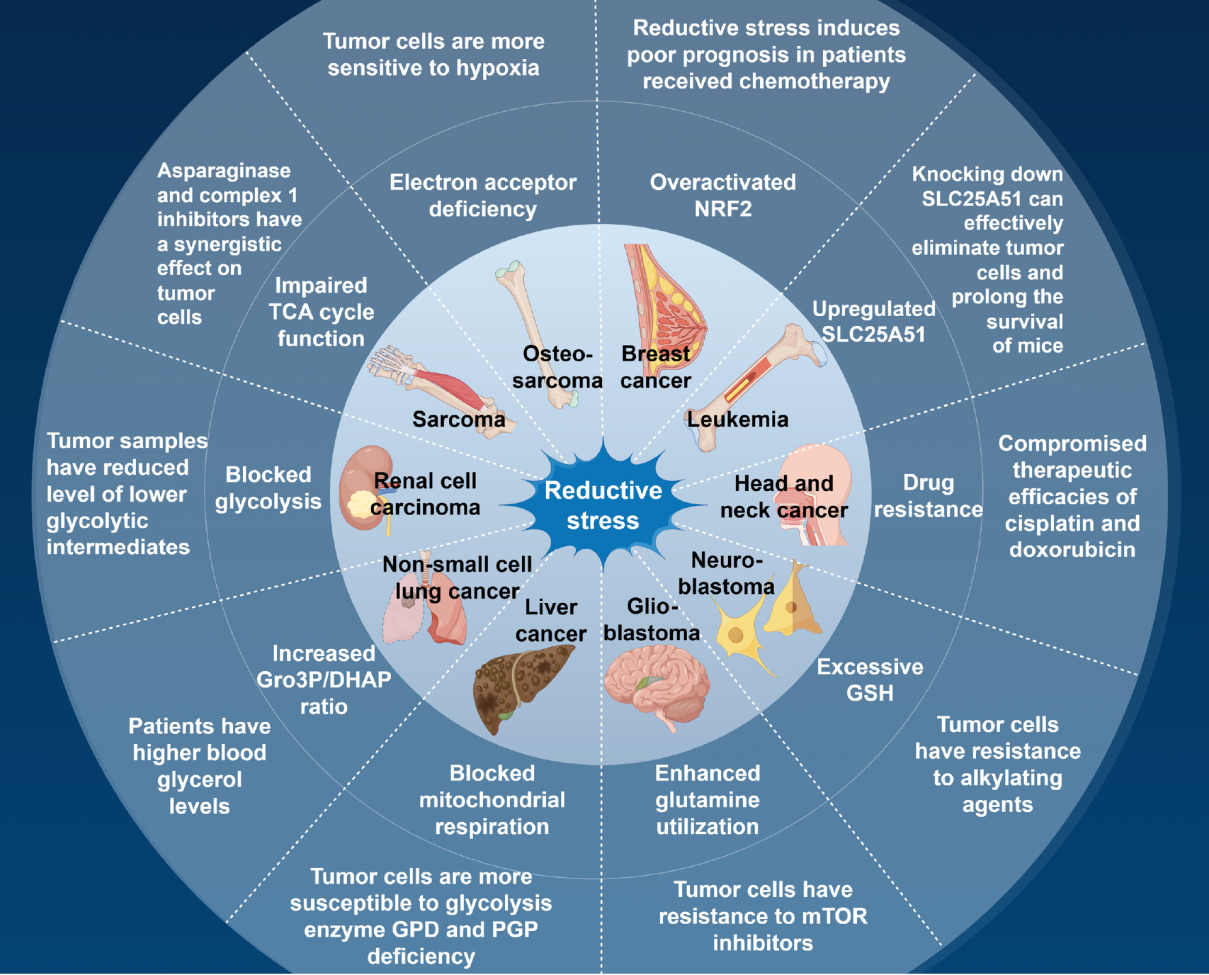
Figure 3 Reductive stress in tumor cells Reductive stress can widely bring impact to many metabolism pathways in tumor cells. It may inhibit cancer development, even eliminate tumor cells, and contribute to drug resistance sometime. Figure was created by Figdraw (https://www.figdraw.com).

Hypoxia can hinder mitochondrial respiration, reducing the consumption of NADH by the ETC. In the glycolytic process, the intermediate fructose-1,6-bisphosphate (FBP) is converted into DHAP and glyceraldehyde 3-phosphate (G3P) under the action of aldolase A. Under redox-balanced conditions, DHAP undergoes rapid enzymatic recycling to G3P, thereby sustaining glycolytic flux through substrate-level maintenance. However, Zhai *et al*. [70] reported that in various tumor cells, such as lung and liver cancer cells, under hypoxic conditions, mitochondrial respiration is blocked, and the NADH/NAD ^+^ ratio increases. The resulting reductive stress activates an alternative metabolic branch of glycolysis, where DHAP is converted to Gro3P by GPD, consuming NADH and alleviating reductive stress. In clear cell renal cell carcinoma (ccRCC) under hypoxic conditions, HIF-1α reduces mitochondrial oxygen consumption, and the cell undergoes reductive stress. Moreover, NAD ^+^-dependent GAPDH has decreased activity, and glycolysis cannot proceed smoothly, leading to impaired cell proliferation ( Figure 3). If exogenous pyruvate is added to act as an electron acceptor, it can consume excess NADH for NAD ^+^ production, thereby restoring GAPDH activity and resuming tumor growth [71]. Similarly, osteosarcoma cells are sensitive to reductive stress caused by hypoxia. Supplementation with the LDH substrate α-ketobutyrate (α-KB) can decrease the intracellular NADH/NAD ^+^ ratio and restore proliferative activity [72]. α-KB, as a substitute for pyruvate, cannot provide carbon atoms or produce ATP for cells; it merely acts as an electron acceptor to consume NADH and regenerate NAD ^+^ to alleviate reductive stress, thereby restoring the proliferative activity of osteosarcoma cells. These findings further prove that the inhibition of osteosarcoma cell proliferation caused by hypoxia is closely related to reductive stress.

Another characteristic that distinguishes tumor cells from somatic cells is that tumor cells often distort their nutrient utilization for aberrant proliferative activities. Nonessential amino acids are a class of amino acids that cells can synthesize *de novo*. However, after metabolic reprogramming, many non-essential amino acids become nutrients that tumor cells depend on for survival. This unique amino acid metabolism can also disrupt cellular redox homeostasis. Asparagine depletion has now become an important clinical cancer treatment strategy. Bauer *et al*. [73] reported that sarcoma cells with asparagine deficiency are under reductive stress. In the absence of an exogenous source, tumor cells synthesize asparagine by converting glutamine to aspartate, leading to the continuous accumulation of NADH synthesized in the TCA cycle, an increased NADH/NAD ^+^ ratio, and impaired TCA cycle function due to the lack of electron acceptors, which impedes tumor cell proliferation. Simultaneously, inhibiting mitochondrial ETC complex I (which consumes NADH to regenerate NAD ^+^) with agents while depleting asparagine can exacerbate reductive stress in sarcoma cells, further inhibit proliferation, and induce apoptosis.

When intracellular antioxidant genes are dysregulated in tumor cells, excessive accumulation of reducing equivalents occurs, leading to reductive stress. Compared with adjacent somatic cells, breast cancer cells have higher levels of reduced thioredoxin, resulting in reductive stress [74]. There is also a small subset of cancer stem cells (CSCs) in breast cancer that have stem cell characteristics. CSCs are known to have self-renewal and differentiation abilities, driving cancer recurrence and drug resistance. Kim *et al*. [75] reported that the antioxidant factor NRF2 in breast cancer CSCs was hyperactivated and upregulated the expression of glutamate-cysteine ligase catalytic subunit (GCLC), resulting in the massive synthesis of GSH and reductive stress. The transporter protein SLC25A51 is responsible for regulating redox homeostasis in mitochondria. When excess NADH accumulates in mitochondria, SLC25A51 pumps oxidized NAD ^+^ from the cytoplasm into the mitochondrial matrix to counteract fluctuations in the NADH/NAD ^+^ ratio and sustain cellular respiration. Lu *et al*. [76] reported that acute myeloid leukemia (AML) cells significantly upregulate SLC25A51 expression to counteract the reductive stress caused by excess NADH in mitochondria ( Figure 3). Moreover, knocking down *SLC25A51* in AML cells increased the NADH/NAD ^+^ ratio in mitochondria, leading to reductive stress, which can effectively eliminate AML cells and significantly prolong the survival of mice. Vellama *et al*. [77] reported that ccRCC cells with von Hippel-Lindau (VHL) gene defects presented aberrant HIF-1α accumulation under normoxia, with a relatively high NADH/NAD ^+^ ratio, impaired cellular respiration and TCA cycle function, and reductive stress manifested as increased reductive carboxylation of glutamine.

Fundamentally, tumor cells exhibit heightened susceptibility to redox imbalance due to microenvironmental hypoxia, pathological metabolic remodelling, and oncogenic alterations. Consequently, many malignancies manifest constitutive reductive stress signatures of varying intensity ( Figure 3). Reductive stress typically inhibits tumor progression via its broad effects on cellular processes, although it may occasionally facilitate cancer survival. However, in some cases, it has been found to facilitate resistance to treatment and relapse in tumor cells.

## Reductive Stress and Tumor Drug Resistance

Despite significant advances in cancer treatment during recent decades, drug resistance remains an inevitable challenge. As mentioned earlier, tumor cells often exhibit shifted redox homeostasis due to metabolic reprogramming, and a reductive intracellular environment has been reported to confer drug resistance.

Many chemotherapeutic drugs eliminate tumor cells by inducing the accumulation of ROS and oxidative stress. When tumor cells exist in a reducing intracellular environment, excessive reducing equivalents can hinder the ability of chemotherapeutic drugs to increase ROS levels, leading to drug resistance. Kasherman *et al*. [78] reported that non-small cell lung cancer with resistance to cisplatin presented increased levels of GSH. Current research suggests that GSH can bind to cisplatin molecules to form intermediate products that eliminate their toxicity. However, the binding efficiency between the two is not high, so further investigation is needed to understand the resistance of cisplatin caused by high levels of GSH. Similar cases include the elevated expression of glutathione S-transferase (GST) in head and neck cancer and breast cancer, which leads to the conjugation of GSH to cisplatin and doxorubicin, respectively, compromising chemotherapeutic efficacy [ 79, 80] ( Figure 3).

Glutamine, a nonessential amino acid, is an important nutrient for the survival of many tumor cells. It provides a carbon source for the TCA cycle and is used to synthesize other nonessential amino acids, nucleotides, and lipids. Glutamate, which is converted from glutamine, is one of the precursor amino acids for GSH. Therefore, high-intensity glutamine metabolism in tumor cells may increase GSH levels, leading to high reduction potential and drug resistance. Izaki *et al*. [59] reported that neuroblastoma cells with high levels of reductants and a reducing intracellular environment cannot be effectively killed by alkylating agents such as melphalan because GSH can bind to drug molecules and transport them out of the cell to resist drug toxicity. Restricting glutamine uptake in neuroblastoma cells can decrease GSH levels and overcome this resistance. Similarly, Tanaka *et al*. [60] reported that when mTOR inhibitors (such as rapamycin) are used to treat glioblastoma multiforme cells, the expression level of glutaminase (GLS1) in tumor cells increases, increasing glutamine utilization and resisting the toxicity of mTOR inhibitors.

The overexpression of antioxidant genes in tumor cells can also confer resistance to chemotherapy. Wang *et al*. [81] reported that during the treatment of acute B lymphoblastic leukemia (B-ALL) with vincristine (VCR), the sensitivity of tumor cells to VCR is often related to the expression level of the key transcription factor NRF2. The efficacy of VCR is lower in patients with high NRF2 expression, indicating that the highly reducing state caused by NRF2 expression confers resistance to VCR in B-ALL cells.

In summary, tumor cells with excessive reducing equivalents are more resistant to chemotherapies, especially those that induce ROS accumulation. Consequently, malignancies exhibiting heightened reductive potential necessitate systematic characterization of their metabolic dependencies to identify targetable vulnerabilities, thereby enabling precise therapeutic interventions.

## Cancer Therapy Based on a Reductive Stress-enhancing Strategy

Given the pathophysiological consequences of reductive stress in tumor cells, therapeutic exacerbation of redox imbalance beyond cellular tolerance thresholds represents a rational strategy for eliminating therapy-refractory tumors. The efficacy of reductive stress-inducing treatment depends on the basal redox state and metabolic patterns of tumor cells. Thus, it is crucial to identify the genetic or metabolic profiles that could indicate the sensitivity of certain tumor cells to that intervention.

Agents that block aerobic respiration, which oxidizes NADH, can exacerbate reductive stress in tumor cells and achieve a significant cytotoxic effect. Metformin, a mitochondrial electron transport chain complex I inhibitor, has been widely used in cancer treatment. Gui *et al*. [82] reported that metformin treatment induced metabolic reprogramming in lung cancer cells, characterized by an elevated NADH/NAD⁺ ratio, impaired TCA cycle flux, and consequential reductive stress that mediated proliferation arrest. If additional electron acceptor pyruvate is supplemented to consume NADH and regenerate NAD ^+^, thereby alleviating reductive stress, metformin cannot exert its cytotoxic function. In addition to the mitochondrial ETC for aerobic respiration, mammalian cells also have a plasma membrane electron transport (PMET) pathway that can consume oxygen on the cell membrane surface and oxidize cytoplasmic NADH for respiration. Blocking the PMET process can exacerbate the reductive stress toxicity within the cell, which is especially effective for tumor cells that rely on PMET to regulate reductive stress and achieve rapid proliferation [83] ( Figure 1).

The activity of the transcription factor NRF2 accounts for the formation of a cellular environment with increased reduction potential. Therefore, it is possible to predict whether tumor cells are under reductive stress on the basis of NRF2. The persistent activation of NRF2 in B-ALL cells leads to the accumulation of reducing equivalents, causing reductive stress and conferring resistance to conventional chemotherapeutic drugs such as VCR, Ara-C, and doxorubicin [81]. Kannan *et al*. [84] reported that activating the Notch pathway can decrease the expression level of NRF2 in AML and overcome doxorubicin resistance by alleviating the reductive state of the cells, similar to the effect of the NRF2 inhibitor brusatol. Chen *et al*. [85] used near-infrared (NIR) light irradiation combined with special nanoparticles for tumor cell treatment. After NIR irradiation, the nanoparticle shell releases electrons to disrupt the original oxidative microenvironment of the endoplasmic reticulum and activate the NRF2 system, leading to reductive stress that interferes with protein folding and induces apoptosis in tumor cells. Weiss *et al*. [86] reported that when lung tumor cells had mutations in the NRF2 inhibitory protein KEAP1, tumor cells were more vulnerable to ETC complex I inhibitors. Treating *KEAP1*-wild-type lung cancer cells with KEAP1 inhibitors can also increase the sensitivity of tumor cells to ETC complex I inhibitors. In the same study, when KEAP1 expression was defective or inhibited, activated NRF2 upregulated the expression of the aldehyde dehydrogenase ALDH3A1, increasing the NADH/NAD ^+^ ratio. Combining with ETC complex I inhibitors further exacerbates reductive stress and significantly eliminates tumor cells. Hyperactivation of the NRF2 pathway increases the cellular reductive potential but paradoxically fails to uniformly induce apoptosis in tumor cells, particularly those exhibiting a constitutively oxidized baseline redox status. In contrast to tumor elimination, a reductive cellular environment can confer resistance to conventional chemotherapeutic drugs in tumor cells. Moreover, the reductive stress caused by NRF2 also promotes the generation of a tumor immunosuppressive microenvironment, reducing the infiltration of immune cells. Therefore, the design of a reductive stress-inducing strategy and the identification of efficient biomarkers for cancer treatment could be delicate because of the potentially narrow therapeutic window.

In addition to the KEAP1-NRF2-mediated antioxidant response, other proteins involved in redox balance in tumor cells can also be regarded as therapeutic targets. Noch *et al*. [87] reported that glioblastoma multiforme (GBM) is particularly sensitive to the antioxidant compound NAC compared with normal neuronal cells and other tumor cells. NAC treatment can significantly decrease the mitochondrial oxygen consumption and proliferative activity of GBM. When combined with glucose starvation therapy, it can significantly eliminate GBM cells. When NAC induces reductive stress by depleting oxidized electron acceptors, rapid mitochondrial H _2_O _2_ production in GBM cells is consequently induced, leading to eventual cytotoxicity. Analysis of the Clinical Proteomic Tumor Analysis Consortium (CPTAC) database revealed that GBM has lower expression levels of redox enzymes (TrxR2, GR). These findings indicate that GBM cells cannot effectively maintain intracellular redox homeostasis; thus, GBM cells are more sensitive to NAC. As mentioned earlier, the transporter SLC25A51 can pump cytoplasmic NAD ^+^ into mitochondria to maintain the balance of the mitochondrial NADH/NAD ^+^ ratio. Lu *et al*. [76] reported that knocking down *SLC25A51* expression in AML cells promoted the sensitivity of AML cells to conventional chemotherapeutic drugs such as azacitidine (a DNA methylation inhibitor). Previous studies reported that the oxidized thioredoxin Trx disulfide in mammalian cells can bind to NLRP1 and inhibit inflammasome formation. When the radical-trapping antioxidant JSH-23, which converts oxidized Trx to reduced Trx, is used to treat tumor cells, the inflammasome can be easily activated, leading to pyroptosis. Notably, macrophages are also affected by this mechanism [ 52, 53] .

To precisely predict the sensitivity of tumor cells to reductive stress, key metabolic activities or biomarkers that account for the formation of a reductive cellular environment in that particular tumor type must be identified. Through metabolomics analysis, Halbrook *et al*. [88] reported that a subpopulation of mouse pancreatic tumor cells with a high NADH/NAD ^+^ ratio and low aspartate content is more sensitive to drugs that induce reductive stress, such as mitochondrial inhibitors (oligomycin, phenformin), lactate dehydrogenase inhibitors (FX11), and transaminase inhibitors (AOA). Notably, if asparagine is used to pretreat these subpopulations, the intracellular aspartate content increases, and cells acquire resistance to the ETC inhibitor oligomycin. In a mouse model of pancreatic cancer, a ketogenic diet can partially increase the content of NADH. However, combined treatment with triple-agent chemotherapy (paclitaxel/gemcitabine/cisplatin) significantly increases the NADH/NAD⁺ ratio, augmenting reductive stress to suppress tumorigenesis and, notably, prolong overall survival [89]. It has also been reported that a ketogenic diet can promote the infiltration ability of macrophages, but whether it affects the overall outcome of tumor control is still unclear [48].

Several compounds have been reported to directly induce reductive stress and eliminate tumor cells ( Table 1). Treating human non-small cell lung cancer cells with 2,2′-bipyridine diselenide (Py _2_Se _2_) resulted in significant redox modulation, characterized by reduced intracellular ROS levels and an elevated GSH/GSSG ratio. This reductive shift inhibits cellular proliferation, induces endoplasmic reticulum stress, and compromises mitochondrial function, ultimately activating the intrinsic apoptotic pathway to eliminate tumor cells [90]. Gandhi *et al*. [91] reported that 3,3′-diselenoddipropionic acid (DSePA) can significantly eliminate hypoxic human lung cancer cells without affecting somatic cells. After DSePA treatment, the ROS levels in tumor cells decreased, while the GSH/GSSG and NADH/NAD ^+^ ratios increased, leading to apoptosis. A transhydride complex synthesized from platinum complexes can increase GSH levels in neuroblastoma and leukemia cells, inducing reductive stress and inhibiting tumor cell proliferation [92]. Pan *et al*. [93] reported that treating hepatocellular carcinoma model mice with selenite (Na _2_SeO _3_) significantly increased the levels of NADPH and GSH in liver cancer cells and generated large amounts of hydrogen selenide, leading to reductive stress and ultimately killing tumor cells through the autophagy pathway. Gao *et al*. [94] reported that treating hypoxic hepatocellular carcinoma cells with the antioxidant ascorbic acid can significantly induce apoptosis in tumor cells. Magnetite nanoparticles (Fe _3_O _4_ NPs) are biocompatible nanomaterials that are convenient for drug delivery and have been used in the clinical treatment of various diseases. Lewińska *et al*. [95] reported that when magnetite nanoparticles are coated with an amorphous carbon shell based on glucosamine (Fe _3_O _4_@aC), they exhibit strong reducing activity. The administration of these nanoparticles to breast cancer cells decreased intracellular ROS levels, further increasing the expression levels of the antioxidant proteins FOXO3a, SOD1, and GPX4. This coordinated redox shift induced a reductive state that synergistically enhanced the cytotoxicity of the conventional chemotherapeutic agent etoposide.

**Table:** **Table 1** Overview of drugs in cancer therapy that target reductive stress

Drug	Mechanism of action	Note	Ref.
Metformin	Mitochondrial electron transport chain complex I inhibitor	Inhibited lung cancer proliferative activity with increased NADH/NAD ^+^ ratio; FDA approved in type 2 diabetes	[82]
NIR with nanoparticle	The nanoparticle shell release electrons to activate NRF2	Leaked electrons induce reductive stress of breast cancer cell, resulting in misfolded protein and apoptosis	[85]
KI696	Inhibitor of KEAP1-NRF2 interaction	Overactivated NRF2 upregulates the expression of ALDH3A1, increasing NADH/NAD ^+^ ratio, turning out non-small cell lung cancer cells more vulnerable to ETC complex I inhibitors	[86]
N-acetylcysteine (NAC)	Antioxidant drug	Reduced GBM cells’ mitochondrial oxygen consumption and proliferative activity; FDA approved in dietary supplements	[87]
Oligomycin\phenformin\FX11\AOA	Mitochondrial ETC inhibitors\LDH inhibitor\transaminase inhibitor	Exhibited more effective to a subpopulation of mouse pancreatic cancer cells with a high NADH/NAD ^+^ ratio and low aspartate	[88]
2,2′-bipyridine diselenide (Py _2_Se _2_)	Increases the GSH/GSSG ratio	Induced ER stress, impaired mitochondrial function, and apoptosis of non-small cell lung cancer cells	[90]
3,3′-diselenoddipropionic acid (DSePA)	Increases the GSH/GSSG and NADH/NAD ^+^ ratio	Induced apoptosis of hypoxic lung cancer cells	[91]
A trans-hydride complex synthesized from platinum	Increased GSH levels	Inhibited proliferation of neuroblastoma and leukemia cells	[92]
Na _2_SeO _3_	Generated large amounts of hydrogen selenide	Increased the levels of NADPH and GSH in hepatocellular carcinoma model mice, and killed cancer cells through autophagy pathway	[93]
Ascorbic acid	Decreased ROS	Induced apoptosis in hypoxic hepatocellular carcinoma cells	[94]
Magnetite nanoparticle (Fe _3_O _4_@aC)	Increased the expression levels of antioxidant proteins FOXO3a, SOD1, and GPX4	Exerted significant combined cytotoxic effect with etoposide on breast cancer cells	[95]

In summary, reductive stress can be leveraged to target tumor cells for therapy, although sometimes cancers can exploit it to confer drug resistance. Considering that diverse strategies have been used by tumor cells to reprogram metabolic pathways for progression, identifying specific treatment responses, metabolic patterns and genetic lesions that inform the preference of redox states and sensitivity to reductive stress-enhancing treatment is vital.

## Conclusions and Perspectives

Electron donors and acceptors participate in all aspects of cellular energy metabolism and biomacromolecule synthesis. Their interconversion maintains the intracellular redox balance. However, excessive accumulation of either can cause oxidative or reductive stress, hindering cellular function and even survival. Many studies have revealed the molecular mechanisms of oxidative stress in mediating cellular damage, but the role of reductive stress, which has a more complex impact, remains elusive. Unlike oxidative stress, which can be defined by the overaccumulation of intracellular ROS, reductive stress is caused by excessive electron donors. Pileup of diverse electron donors can influence and damage cellular metabolism and proliferation through various pathways. For example, excessive GSH and NADPH reduce ROS levels and disrupt signaling and protein disulfide bond formation, leading to abnormal protein aggregation. However, too much NADH mainly causes mitochondrial dysfunction, and reduced flavoproteins may leak electrons to produce ROS, paradoxically turning reductive stress into oxidative stress for further damage.

Electron donors are closely linked to cellular biosynthetic activities. Cells with high biomass and proliferative demands, such as immune and tumor cells, are more susceptible to reductive stress. When immune cells respond to tumors, their redox state shifts with activation, proliferation, and differentiation. Reductive stress can simultaneously inhibit effector T cells and regulatory T cells. It also enhances macrophage infiltration but affects proinflammatory factor synthesis differently in the context of the TME. Thus, the impact of reductive stress on the overall immune system does not follow a simple working model. Tumor cells, which exhibit unrestricted proliferation, metabolic reprogramming, and redox imbalance, can be selectively killed by pharmacologically induced reductive stress. Furthermore, these cells may develop resistance to certain ROS-inducing treatments and promote an immunosuppressive microenvironment, resulting in resistance and relapse.

This review critically re-examines the conceptual framework of reductive stress and synthesizes its mechanistic implications for cancer-immune dynamics while concurrently highlighting unresolved questions: What molecular determinants (including lineage specificity, transcription profiles, signaling dependency and metabolic programs) confer tumor type-specific variation in reductive stress susceptibility? How does reductive stress manifest divergent immunomodulatory effects? Which biomarker could effectively predict the therapeutic sensitivity of patients to reducing-stress-enhancing treatment?

A comprehensive investigation of these fundamental questions is necessary. This review aims to investigate reductive stress within cancer-immune crosstalk in immune-competent contexts and its potential synergism with immunotherapeutic modalities. We posit that systematic elucidation of the mechanisms regulating reductive stress will reveal context-dependent regulatory paradigms governing cancer-immune interactions. To date, several hypothetical frameworks have emerged. (1) The tumor sensitivity hypothesis is as follows: elevated reducing-equivalent amounts caused by specific genetic mutations or transcription programs may stratify tumors according to vulnerability to reducing-stress-enhancing treatment. (2) Immunomodulatory paradox: divergent outcomes of reductive stress in the immune response likely arise from tumor type-specific remodeling of the immune microenvironment. (3) Precise intervention principle: reductive stress exhibits context-dependent efficacy—the overall therapeutic benefit requires precise spatiotemporal control to fine-tune the tumor-immune cell interaction. Mechanistic studies providing a better understanding of redox hemostasis could enable rational design of therapies that selectively eliminate malignant cells while reprogramming immunosuppressive TME networks through controlled reductive stress potentiation.
